# Vertical Stratification Drives Divergent Spatial Trade‐Offs Among Xylem Cell Types in Angiosperm Trees of a Mountain Forest in Eastern China

**DOI:** 10.1002/ece3.72916

**Published:** 2026-01-07

**Authors:** Qihang Yang, Yuxin Hong, Hugh Morris, Faguang Pu, Zuhua Song, Xijin Zhang, Kun Song

**Affiliations:** ^1^ Zhejiang Tiantong Forest Ecosystem National Observation and Research Station, School of Ecological and Environmental Sciences East China Normal University Shanghai China; ^2^ School of Natural and Social Sciences SRUC Barony Dumfries UK; ^3^ Anhui Dabie Mountain Forest Ecosystem National Observation Station Lu'an China; ^4^ Key Laboratory of National Forestry and Grassland Administration on Ecological Landscaping of Challenging Urban Sites Shanghai Academy of Landscape Architecture Science and Planning Shanghai China; ^5^ Institute of Eco‐Chongming Shanghai China

**Keywords:** fiber, forest structure, functional trait, parenchyma, vessel, wood anatomy

## Abstract

Vertical stratification in forests acts as an ecological filter, driving woody plants to evolve specialized survival strategies. Angiosperms, in particular, develop secondary xylem with three interdependent functions—water transport, mechanical support, and storage. Trade‐offs between these functions vary with resource heterogeneity and environmental pressures. Balancing these functions is based on trade‐offs in xylem structure, particularly in the xylem space allocation of vessels, fibers, and parenchyma fractions. However, how plants optimize these trade‐offs along forest vertical strata remains unexplored. Anatomical methods were used to determine the fractions of vessels, fibers, and parenchyma in the secondary xylem of 119 individuals within a multilayered forest in eastern China. Ternary plots and standardized major axis analyses were employed to evaluate variations in trade‐offs between vessel and fiber fractions, and between parenchyma and fiber fractions across different vertical strata. We found that trade‐offs in spatial allocation among cell types occur in all vertical strata. For the fiber—vessel trade‐off, canopy and understory trees followed a similar pattern, but canopy trees consistently maintained a higher vessel fraction. In contrast, the fiber—parenchyma trade‐off was markedly stronger in understory trees. Our results illustrate that forest vertical stratification significantly influences trade‐offs in xylem cell allocation, suggesting functional trade‐offs of xylem depend on forest strata. These findings will help clarify how trees adapt to stresses associated with vertical forest strata.

## Graphical abstract

To understand how vertical forest stratification influences xylem functional balance, we measured vessel, fiber, and parenchyma fractions across 39 tree species in eastern China. Key trade‐offs exist between vessel‐fiber and parenchyma‐fiber fractions. For a given fiber fraction, canopy trees allocate more space to vessels, reflecting heightened hydraulic demand. In contrast, understory trees exhibit a stricter, near one‐to‐one trade‐off between fiber and parenchyma (divergent growth strategies).

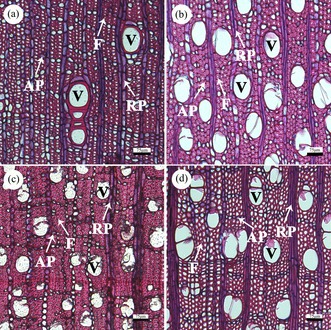

## 1 Introduction

The vascular system in woody plants is structurally complex, with various cell types performing crucial roles such as hydraulic transport, mechanical support, and storage of nonstructural carbohydrates (NSC) (Chave et al. 2009). In particular, the secondary xylem of angiosperms typically comprises three essential cell types: vessels, parenchyma, and fibers (Hellmann et al. 2018). Vessels, as the primary water‐conducting cell type in angiosperms, are essential for water transport (Tyree and Ewers 1991; Chave et al. 2009; Dória et al. 2022). Parenchyma consists of living cells and serves as the principal tissue for the storage of nonstructural carbohydrates. It is further categorized into ray parenchyma and axial parenchyma based on their arrangement and orientation (Spicer 2014; Morris et al. 2016; Plavcová et al. 2016; Słupianek et al. 2021). Fibers play critical roles in providing mechanical support and water capacitance (Hacke et al. 2001; Ziemińska et al. 2013; González‐Melo et al. 2025). As vessels, parenchyma, and fibers share a finite xylem space, an increase in the proportion of one cell type can reduce the space available for other cell types and their associated functions (Morris et al. 2016; Pratt, Jacobsen, et al. 2021). The trade‐offs among proportions of different cell types are the structural basis for the functional diversity of xylem (Pratt, Jacobsen, et al. 2021). Resource allocation within xylem space may involve functional trade‐offs, where xylem tissue specialized in one functional area may be less proficient in another (Preston et al. 2006; Sperry et al. 2006; Pratt et al. 2007; Zhang, Yang, et al. 2024). Therefore, elucidating how angiosperm trees adjust xylem functions through trade‐offs among different cell types to cope with stress and potentially maximize fitness is crucial for understanding the physiological and morphological diversity of woody plants (Pratt and Jacobsen 2017).

Trade‐offs among xylem cell types exhibit considerable variation among woody angiosperms and vary across different climate zones. Under tropical climates, the trade‐offs between fiber and parenchyma fractions tend to be stronger than in temperate climates, whereas the trade‐offs between vessel and fiber fractions are more pronounced under temperate climates (Morris et al. 2016; Zheng et al. 2019; Zhang et al. 2023). These trade‐offs also vary among different growth forms. For example, Pratt and Jacobsen (2017) reported strong and negative correlations between fiber and parenchyma fractions and between fiber and vessel fractions. In contrast, Zhang et al. (2023) found no relationship between fiber and parenchyma fractions across 20 temperate tree species. In summary, clear trade‐offs between the fractions of different xylem tissues remain inconclusive and their relationships likely depend on the biotic environment and plant growth forms.

In forest ecosystems, vertical stratification—comprising distinct layers from the canopy to the forest floor—functions as an ecological filter that selectively influences the survival strategies of woody plants (Xing et al. 2023). To adapt to the stresses and changing demands encountered across different vertical forest strata, trees must maintain balances among different xylem functions, which may affect the spatial allocation of different xylem tissues across forest strata. Specifically, as trees ascend through the canopy layers, they face distinct biological and physical pressures, including shifts in the demands for water transport, mechanical support, and light exposure (Petit et al. 2023). The hydraulic limitation hypothesis predicts that as the length of the water transport pathway in the xylem increases, hydraulic conductivity gradually decreases (Ryan et al. 2006; Lechthaler et al. 2020; Petit et al. 2023). Consequently, taller canopy trees typically develop wider xylem vessels to compensate for reduced hydraulic conductivity (Ryan and Yoder 1997; Koch et al. 2004; Araújo et al. 2024). However, wider vessels may also increase susceptibility to embolism compared to the narrower vessels typically of understory shorter trees (Olson et al. 2018; Liu et al. 2019; Lens et al. 2022). The presence of parenchyma, especially axial parenchyma in contact with vessels, could promote increased hydraulic capacitance, potentially reducing embolism susceptibility (Morris et al. 2018; Kiorapostolou et al. 2019; Janssen et al. 2020; Ziemińska et al. 2020; Aritsara et al. 2021).

Several studies indicate that taller trees tend to allocate more xylem space to fibers to enhance mechanical support (Pratt and Jacobsen 2017; Rodriguez‐Zaccaro et al. 2019; Angélico et al. 2021). However, research also shows that fiber wall fraction is a crucial determinant of wood density, suggesting that increasing fiber fraction is not necessarily required to improve mechanical support (Ziemińska et al. 2013; Zhang, Yang, et al. 2024). Thus, during the vertical transition from understory small trees to canopy tall trees in forest ecosystems, the requirement for greater mechanical strength and improved hydraulic safety also increases (Wu et al. 2025). Trees adjust their xylem structure to meet these changing functional demands for hydraulic transport, mechanical support, and resource storage, influencing the trade‐offs among cell types (Pratt and Jacobsen 2017). Therefore, trees in different vertical strata likely demonstrate distinct strategies, potentially leading to variations in the spatial trade‐offs within their wood anatomical structure, which remains to be investigated.

To investigate how trade‐offs among different xylem cell types vary across the vertical strata, we collected wood samples from 119 individuals of 39 tree species in a mountain forest of eastern China. These samples spanned different vertical strata from understory to canopy. Anatomical analyses were used to quantify the fractions of vessels, fibers, and parenchyma in the secondary xylem of stems to investigate the trade‐offs in their spatial allocation across different vertical strata. We hypothesized that trade‐offs among vessels, fibers, and parenchyma fractions shift with different forest vertical strata, driven by changes in the relative demands and limitations associated with light availability, water transport, and mechanical support. Specifically, we predicted that in the canopy stratum, where high demands exist for efficient hydraulic conductance and robust mechanical support, the primary trade‐off involves balancing investment between vessels and fibers. Conversely, we expected that in the understory strata, characterized by greater resource limitation, particularly light, and heightened vulnerability to physical disturbances, pests, and pathogen attacks, the dominant trade‐off occurs between fibers and parenchyma.

## 2 Materials and Methods

### 2.1 Study Sites, Species and Sampling

The study was conducted on Mt. Tiantangzhai in Anhui Tianma National Nature Reserve, China. The reserve is located between latitude 30°10′–31°20′ N and longitudes 115°20′–115°50′ E, in the southwestern part of Jinzhai County, Anhui Province, on the northern slope of the Dabie Mountains. The reserve is situated in the North Subtropical region along the middle and lower reaches of the Yangtze River basin, characterized by a humid monsoon climate. The area experiences four distinct seasons, with substantial temperature variations both monthly and annually. The average annual temperature within the reserve is 13.3°C, with an annual mean rainfall of 1489 mm (Liu et al. 2016; Zhang, Wu, et al. 2022). The region harbors a rich diversity of plant species, comprising 1676 species from 655 genera and 155 families (Liu et al. 2016). The forest in the study area exhibits intricate structural complexity with distinct canopy stratification and notable variations in tree height, making it an ideal site for studying the morphological and physiological responses of plants to variations in different vertical strata.

In June 2021, we selected 119 healthy individual trees with a diameter at breast height (DBH) > 5 cm, belonging to 39 angiosperm tree species (the species present in each vertical stratum and the corresponding number of individuals sampled are shown in Table S1). Using a micro‐core sampler (Trephor), we extracted microcore samples approximately 15 mm in length and 2 mm in diameter from the tree trunks at a height of 1–1.3 m above the ground (Wu et al. 2025). These samples were immediately preserved and fixed in 2 mL centrifuge tubes containing FAA fixative solution (formalin‐acetic acid‐alcohol), with appropriate labeling on the tubes. Concurrently, we measured the heights of the sampled trees by using a Vertex Laser Geo (Vertex Laser Geo, Haglof, Sverige). Due to the dense canopy, the laser rangefinder could typically only target the lower visible edge of the crown. For each tree, we therefore measured the distance to this point with the laser and then added a visual estimate of the remaining height to the apex.

### 2.2 Xylem Anatomical Traits

We prepared cross sections approximately 15–20 μm thick using a sliding microtome (WSL core‐microtome, WSL, Birmensdorf, Switzerland). During the slicing process, we gently brushed a small amount of CWG solution (soluble starch: water: glycerol = 10:8:7) onto the microcore surface to minimize cell rupture and deformation (Schneider and Gärtner 2013). The sections were rehydrated through using distilled water, followed by a 2‐min staining procedure with a solution comprising 0.35% w/v safranin (in 50% ethanol) and 0.65% w/v alcian blue (in distilled water). Subsequently, the stained sections were thoroughly rinsed with distilled water and dehydrated through a graded ethanol series (55%, 75%, 95%, 100%) before being permanently mounted using Neo‐Mount (Merck, Darmstadt, Germany) (Plavcová et al. 2016). The sections were then dried at 50°C for 1 h. To enhance the accuracy of xylem tissue identification in cross‐sections, we also prepared radial and tangential sections for each species.

**FIGURE 1:**
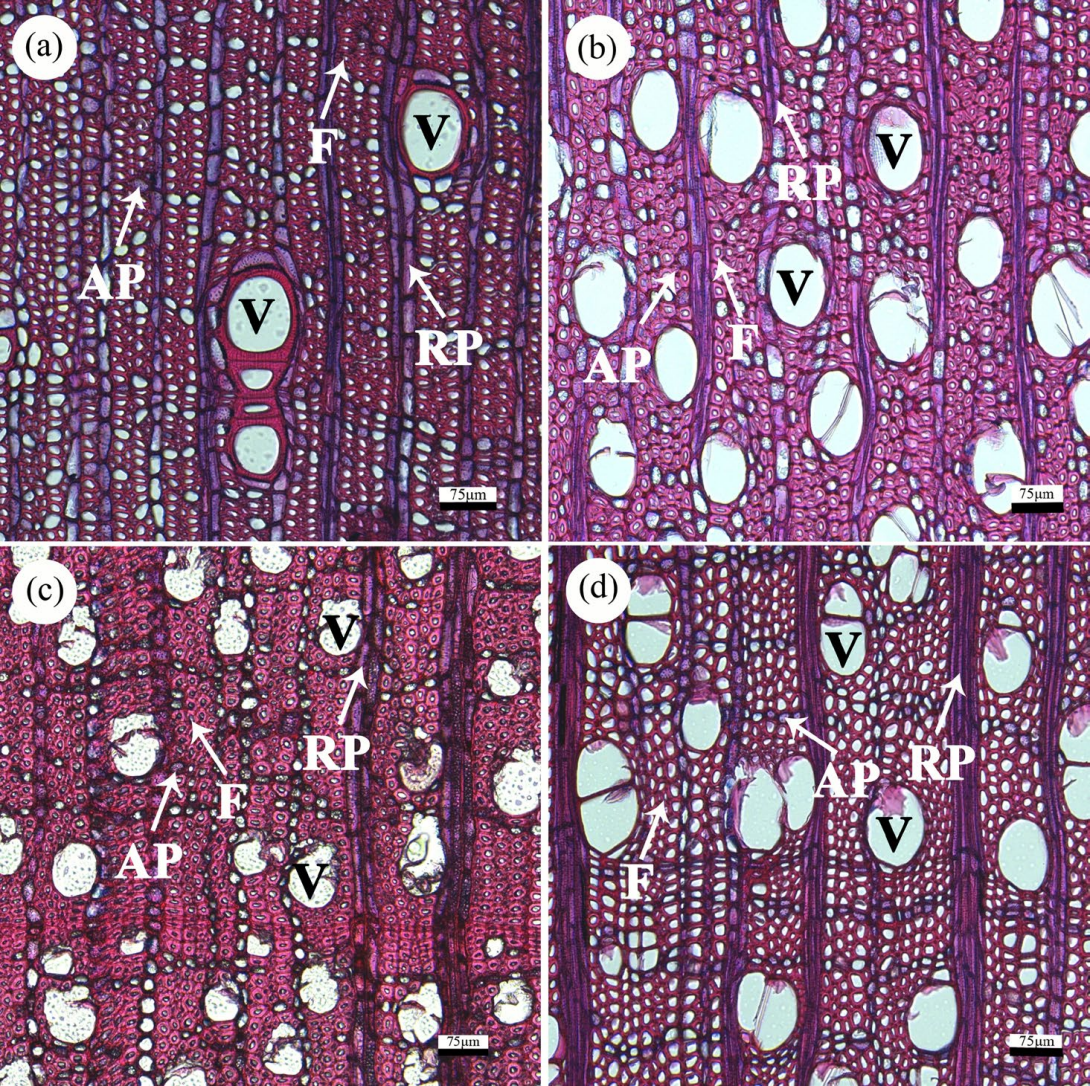
Microscopic images of transverse xylem sections for various species. (a) *Diospyros lotus* ; (b) *Symplocos tanakana*; (c) *Cornus kousa* subsp. *chinensis*; (d) *Styrax obassia*. The sections were stained with a combination of safranin and alcian blue, resulting in a red coloration for strongly lignified cell walls (vessels (V) and fibers (F)) and a blue to purple coloration for both axial parenchyma (AP) and ray parenchyma (RP). All bars, 75 μm.

After placing the sections onto slides, we sequentially photographed them under a microscope (Olympus SZ61, Olympus Corporation, Tokyo, Japan) and stitched the images using PTGui Pro (version 11.12, New House Internet Services BV, Rotterdam, Netherlands) to generate complete cross‐sectional micrographs (Figure 1). We uploaded these micrographs to the CVAT website (https://www.cvat.ai/) and manually delineated specific regions using the polygon annotation tool, ensuring that each delineated area exceeded 2 mm^2^ and encompassed at least one complete annual ring. We utilized distinct colored labels to outline the vessels, axial parenchyma, and ray parenchyma within the delineated regions. For species lacking tracheids, all cells apart from the aforementioned types were identified as fibers (Plavcová et al. 2024). The annotated vessels included both the vessel cell walls and lumens, while the annotated fibers also encompassed the fiber cell walls and lumens. To ensure precise identification of parenchyma tissue types in the cross‐sections, we integrated information from tangential, radial, and cross‐sectional views with image and textual descriptions from the InsideWood database (http://insidewood.lib.ncsu.edu) (Wheeler 2011).

After exporting the annotated image files, R v.4.4.0 was utilized to quantify the fractions of vessels, fibers, and parenchyma and calculated as the ratio of the total area of each xylem cell to the total area of the delineated region.

## 3 Statistical Analyses

To investigate the variations in different cell types with tree height and the potential trade‐offs among vessel‐related traits, we first conducted normality tests on all data. Non‐normal data were log‐transformed to guarantee normality. We then performed simple linear regression analyses to examine the relationships between vessel fraction and fiber fraction, fiber fraction and parenchyma fraction, tree height and vessel diameter, as well as vessel diameter and vessel density. We fitted quadratic models and compared them with linear models using likelihood ratio tests to assess potential nonlinearity in these relationships. Additionally, to explicitly account for species identity, we performed linear mixed‐effects models with species as a random effect.

In order to further clarify the spatial trade‐offs among different vertical strata, we categorized individuals into three forest vertical strata (0–6 m, understory; 6–10 m, subcanopy; 10–16 m, canopy) based on the frequency distribution of tree heights (Figure S1). We used ternary plots in Origin 2021 to illustrate the covariation of vessels, fibers, and parenchyma fractions for all 119 individuals across the three vertical strata. Standardized major axis regression (SMA), a method suitable for analyzing the relationship between two variables that both have error, was used to determine the relationships among the fractions of vessels, fibers, and parenchyma for each vertical stratum (Warton et al. 2012). We calculated *R* ^2^, slopes, intercepts, and their 95% confidence intervals to assess the significance of slopes and intercepts. In the SMA analyses, we assessed slope homogeneity across strata using a likelihood ratio test. If slopes were homogeneous, we then tested for intercept differences using a Wald test. Significant overall differences were followed by Sidak‐adjusted pairwise comparisons. All statistical analyses were conducted in R v.4.4.0, using the “lmodel2” (Legendre and Oksanen 2018) and “smatr” (Warton et al. 2012) packages.

## 4 Results

### 4.1 Tissue Fractions and Their Variations Along Forest Vertical Strata

In our dataset of 119 individuals across 39 species, fibers constituted the highest volumetric fraction of wood at 55.7% (Figure S2). The fiber fraction varied from 36.9% to 80.9%, representing a 2.2‐fold variation (Table S2). Parenchyma occupied 24.3% of wood volume, ranging from 4.4% to 40.1%, representing a 9.1‐fold variation (Figure S2; Table S2). Vessels occupied 20.0% of wood volume and showed the highest variability among wood cell types, ranging from 4.0% to 42.3%, representing a 10.6‐fold variation (Figure S2; Table S2).

**FIGURE 2:**
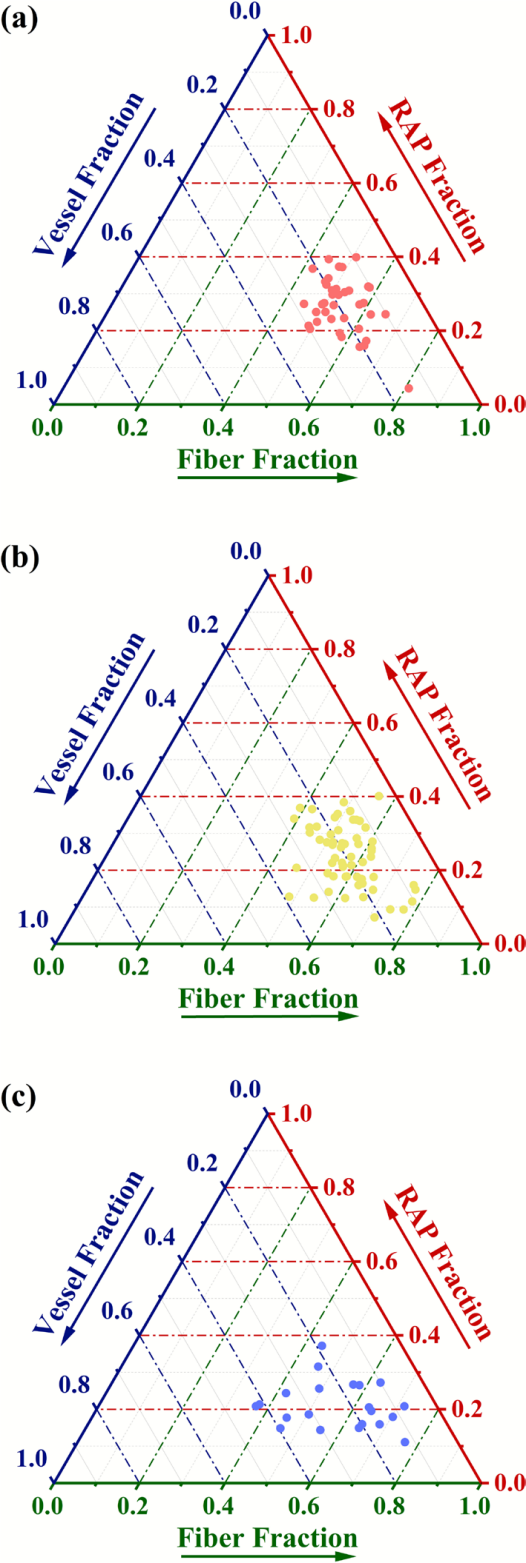
Ternary models of different cell types in wood across different vertical strata individuals. (a) understory, (b) subcanopy, (c) canopy. RAP, total parenchyma fraction includes axial and ray parenchyma.

The distribution of tissue fractions differed notably across vertical strata (Figure 2). For individuals in the understory and subcanopy, fiber fractions primarily concentrated between 0.4 and 0.6, with 18.4% and 31.7%, respectively, exceeding 0.6 (Figure 2a; Figure 2b). However, 42.9% of individuals in the canopy had fiber fractions > 0.6 (Figure 2c). Regarding parenchyma fractions, individuals in the understory and subcanopy primarily ranged between 0.2 and 0.4, with 15.8% and 35.0%, respectively, below 0.2 (Figure 2a; Figure 2b). In contrast, a higher proportion (47.6%) of individuals in the canopy exhibited parenchyma fractions lower than 0.2 (Figure 2c). Vessel fractions for individuals in the understory and subcanopy are primarily concentrated between 0.1 and 0.4, although a minor proportion, 2.6% and 5%, respectively, exhibited vessel fractions below 0.1 (Figure 2a; Figure 2b). In the canopy, 9.5% of individuals displayed vessel fractions below 0.1, while an equal percentage exceeded 0.4 (Figure 2c).

### 4.2 The Overall Trade‐Offs Among Different Tissue Fractions

**FIGURE 3:**
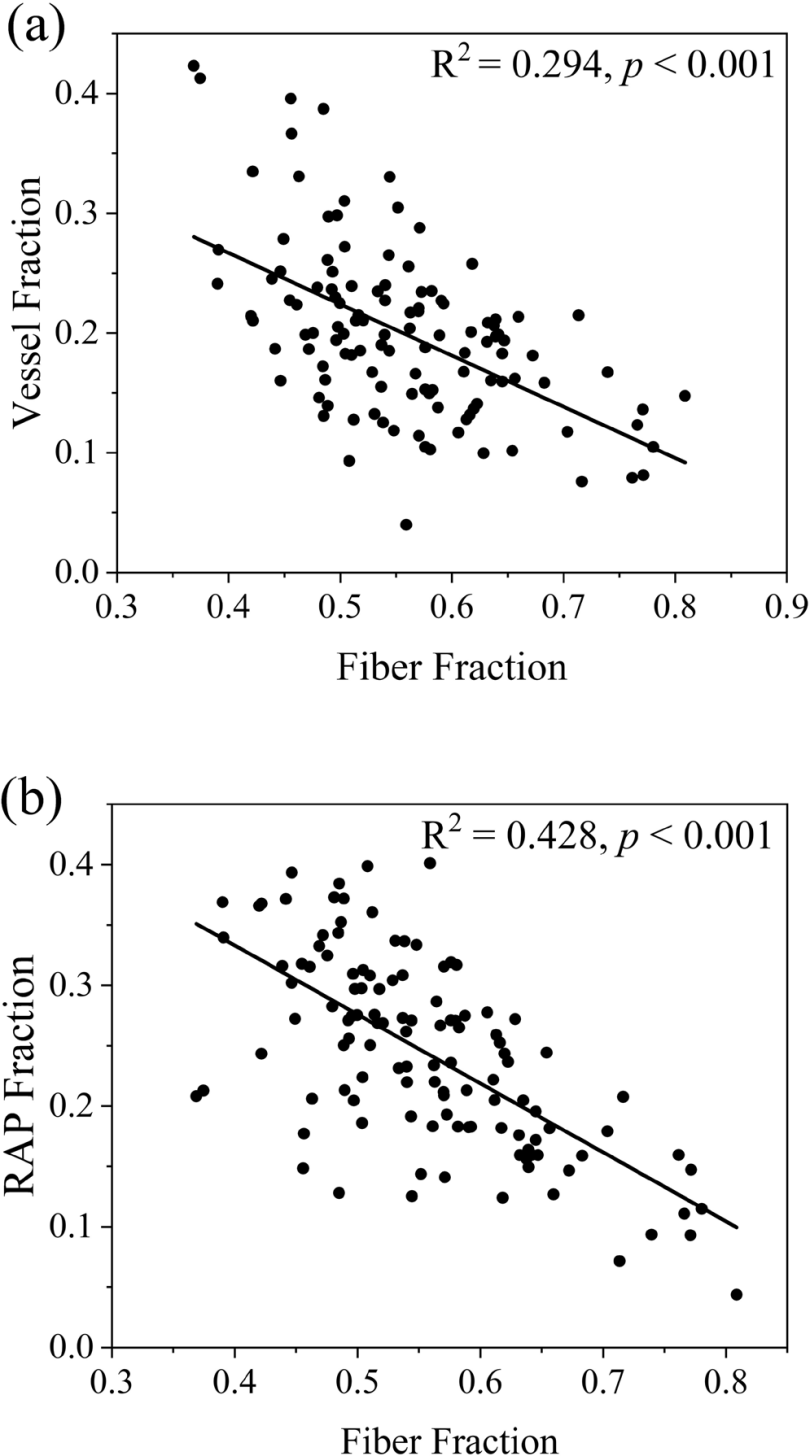
Simple linear regression analyses between pairwise fractions of the three xylem cell types. Significant regression line, *R* ^2^, and *p*‐value are shown. (a) Vessel fraction—fiber fraction, (b) parenchyma fraction—fiber fraction. RAP, total parenchyma fraction includes axial and ray parenchyma.

Across all individuals, a significant negative correlation was observed between fiber fraction and the vessel fraction (*R* ^2^ = 0.294, *p* < 0.001, Figure 3a). Similarly, the parenchyma fraction decreased significantly with increasing fiber fraction (*R* ^2^ = 0.428, *p* < 0.001, Figure 3b). Comparison of linear and quadratic models confirmed the adequacy of linear relationships, with quadratic terms being nonsignificant in most cases (Table S3). To explicitly account for species identity, species was included as a random effect in linear mixed‐effects models; these relationships remained highly significant (vessel‐fiber: coefficient = −0.669, *p* < 0.001; parenchyma‐fiber: coefficient = −0.714, *p* < 0.001; Table S4).

### 4.3 The Trade‐Offs Among Different Tissue Fractions Across Different Vertical Strata

**FIGURE 4:**
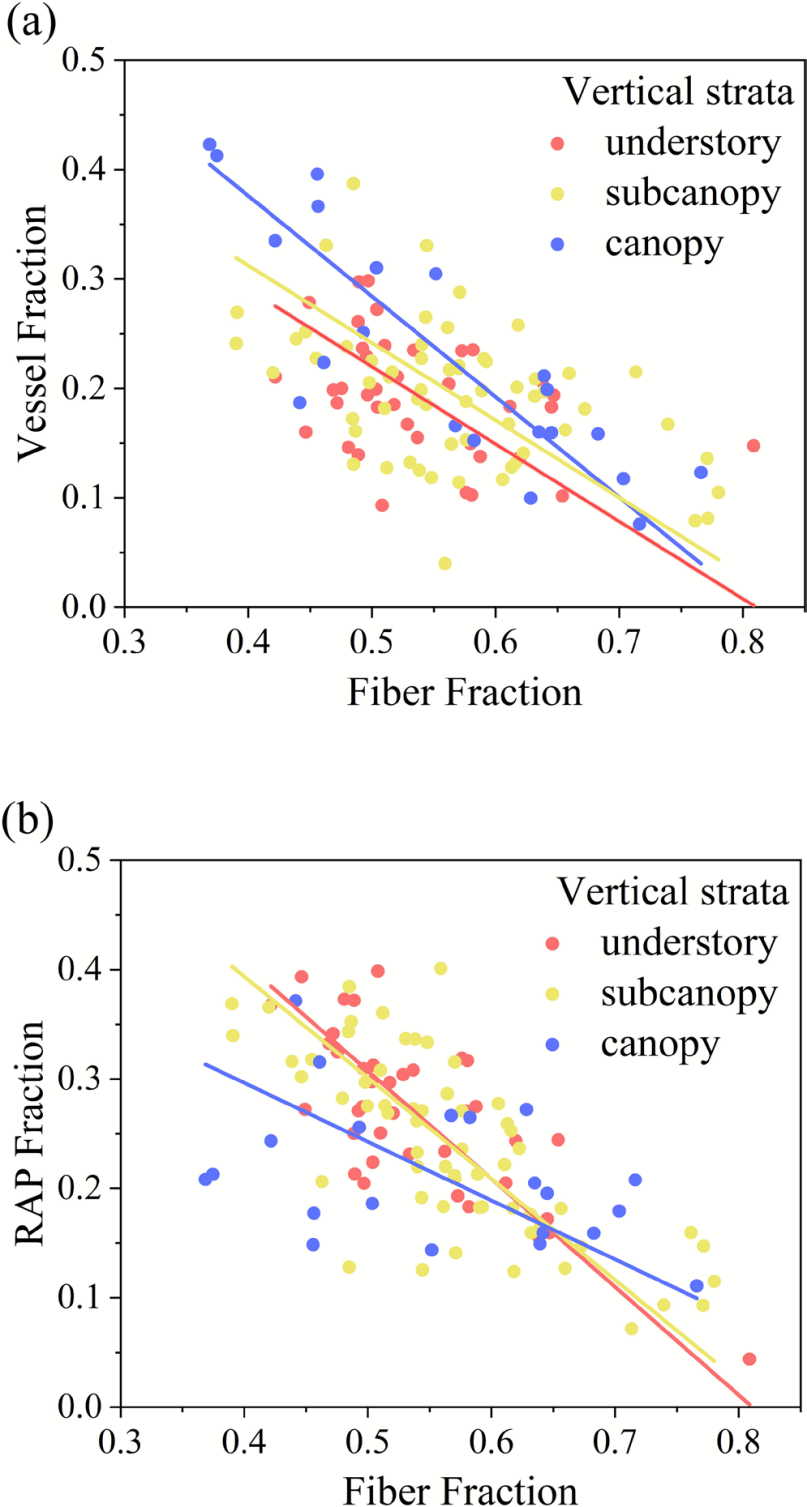
Standardized major axis analyses depicting trade‐offs among different xylem cell types for each vertical stratum. (a) Vessel fraction—fiber fraction, (b) parenchyma fraction—fiber fraction. Model parameters are provided in the accompanying Table 1. Significance of differences in slopes and intercepts among strata is shown in Table 2. RAP, total parenchyma fraction includes axial and ray parenchyma.

For each vertical stratum, there are clear and strong trade‐offs between vessel and fiber fractions, and between parenchyma and fiber fractions (Figure 4). A negative correlation existed between vessel and fiber fractions in all strata (Figure 4a), with slopes of −0.707 (*R* ^2^ = 0.137), −0.707 (*R* ^2^ = 0.207), and −0.918 (*R* ^2^ = 0.715) for individuals in the understory, subcanopy, and canopy, respectively (Table 1). Similarly, the parenchyma fraction was significantly negatively correlated with fiber fraction (Figure 4b), with slopes of −0.988 (*R* ^2^ = 0.558), −0.925 (*R* ^2^ = 0.537), and −0.538 (*R* ^2^ = 0.172) for the understory, subcanopy, and canopy, respectively (Table 1).

**TABLE 1:** Standardized major axis regression slopes and 95% confidence intervals (CI) of the slopes of linear relationships between vessel—fiber fraction and total parenchyma—fiber fraction for different individuals of different vertical strata.

Trade‐offs (*y*‐axis—*x*‐axis)	Vertical stratum	*N*	*R* ^2^	Slope and 95% CI	Intercept and 95% CI
Vessel fraction—fiber fraction	Understory	38	0.137	−0.707 (−0.964, −0.519)	0.574 (0.472, 0.712)
	Subcanopy	60	0.207	−0.707 (−0.891, −0.560)	0.595 (0.512, 0.700)
	Canopy	21	0.715	−0.918 (−1.183, −0.713)	0.743 (0.628, 0.891)
Total parenchyma fraction—fiber fraction	Understory	38	0.558	−0.988 (−1.235, −0.791)	0.802 (0.695, 0.935)
	Subcanopy	60	0.537	−0.925 (−1.105, −0.774)	0.764 (0.678, 0.866)
	Canopy	21	0.172	−0.538 (−0.823, −0.352)	0.512 (0.408, 0.671)

In the fiber—vessel fraction trade‐off relationship, the slopes were homogeneous across three vertical strata (*p* = 0.252; Table 2). However, a significant difference in elevation (intercepts) was detected overall (*p* = 0.006; Table 2). Pairwise comparisons revealed that only the canopy and understory differed significantly in intercept (*p* = 0.004), while no significant differences were observed between other strata (Table 2). For the fiber—parenchyma fraction relationship, the slopes were not homogeneous among strata (overall test: *p* = 0.043; Table 2). Specifically, the slope of the canopy differed significantly from that of the understory (*p* = 0.039), whereas other pairwise slope comparisons were not significant (Table 2). As the slopes were heterogeneous, no elevation (intercept) test was conducted.

**TABLE 2:** Standardized major axis regression analysis of vessel—fiber fraction and total parenchyma–fiber fraction for different individuals across different vertical strata.

Trade‐off relationship	Statistical test	Overall test *p* value	Pairwise comparison results
Fiber fraction—vessel fraction	Slope homogeneity test	0.252	Slopes homogeneous across all three strata
	Elevation difference test	0.006	Understory—subcanopy: *p* = 0.271
			Understory—canopy: *p* = 0.004**
			Subcanopy—canopy: *p* = 0.185
Fiber fraction—total parenchyma fraction	Slope homogeneity test	0.043	Understory—subcanopy: *p* = 0.956
			Understory—canopy: *p* = 0.039*
			Subcanopy—canopy: *p* = 0.062
	Elevation difference test	—	Not tested (inappropriate when slopes differ)

*Note:* *p < 0.05; **p < 0.01, adjusted for multiple comparisons using Sidak method.

## 5 Discussion

### 5.1 Trade‐Offs in Wood Volume Allocated to the Different Cell Types

In our study, analyses indicated significant negative correlations between fiber and vessel fractions and between fiber and parenchyma fractions across vertical strata (Figure 3). These findings support the existence of significant trade‐off relationships involving fiber, vessel, and parenchyma fractions, corroborating findings from numerous studies (Pratt and Jacobsen 2017; Janssen et al. 2020; Dória et al. 2022; Kawai et al. 2022; Zhang et al. 2023). The spatial limitations in wood are a crucial factor underlying the trade‐offs among different wood cell types. This limitation arises because the three primary functions of xylem—material transport, storage, and mechanical support—are structurally supported by fibers, vessels, and parenchyma fractions (Tyree and Ewers 1991; Hacke et al. 2001; Hoch et al. 2003; Deflorio et al. 2008; Chave et al. 2009; Brodersen et al. 2010; Ziemińska et al. 2013; Dória et al. 2022). To maximize fitness in different environments, trees adjust the spatial allocation of these cell types within the limited xylem space, leading to the observed trade‐offs among them (Morris et al. 2016; Pratt and Jacobsen 2017; Pratt, Tobin, et al. 2021; Zhang, Yang, et al. 2024).

The strong negative correlations observed between fiber fraction and both vessel and parenchyma fractions highlight the central role of fiber allocation in shaping the volumetric composition of xylem tissues (Figure 3). The prominence of fiber‐based trade‐offs may be attributed to the central role of fibers in mechanical support, a function less readily substituted by other cell types (Martínez‐Cabrera et al. 2009; Onoda et al. 2010; Plavcová et al. 2019). Previous studies have reported that fiber characteristics associated with wood density and mechanical strength, which are closely related to the general need for enhanced biomechanical performance in canopy stratum and improved resistance to physical disturbances and pest or pathogen attacks (Ziemińska et al. 2013; Plavcová et al. 2019). Specifically, studies such as Ziemińska et al. (2013) have emphasized that fiber wall fraction is a stronger driver of wood density and strength, suggesting that structural modifications at the subcellular level may compensate for variations in tissue area. In contrast, the functions of vessels and parenchyma may be more readily subjected to substitution or influenced by factors beyond their volumetric fraction. For instance, the hydraulic function of vessels can be compensated by wood capacitance mediated parenchyma, which has been shown to achieve a similar physiological effect as high hydraulic conductivity in terms of maintaining water flow (Sperry et al. 2008; Zhang, Li, et al. 2024), particularly in large‐bole trees with significant water storage capacity (Tyree and Yang 1990; Phillips et al. 2003). Furthermore, recent research by Tricerri et al. (2025) suggests that in species with minimal axial parenchyma fractions, water stored in fibers adjacent to vessels may facilitate embolism repair, indicating functional redundancy between cell types. Similarly, the storage role of parenchyma can be partially substituted by living fibers, which have also been shown to store NSC (Plavcová et al. 2016; Pratt, Tobin, et al. 2021; González‐Melo et al. 2025).

Moreover, the functions of vessels and parenchyma are not solely dependent on their fractions but are also influenced by cell size, arrangement, and spatial distribution. For example, hydraulic efficiency is known to be more closely related to vessel diameter and density (Flor et al. 2025). Our observation that vessel diameter increases with tree height (Figure S3), which is consistent with the biomechanical imperative for greater hydraulic conductivity in the canopy, a pattern that also manifests as a trade‐off with vessel density as shown in our Figure S4 (Sperry and Hacke 2004; Choat et al. 2008; Lens et al. 2011; Zhang, Yang, et al. 2024). Likewise, the functional specialization of parenchyma is influenced by its arrangement, with ray and axial parenchyma contributing differently to storage and transport based on their distribution (Zheng and Martínez‐Cabrera 2013; Morris et al. 2018; Plavcová et al. 2024). These findings collectively suggest that while our study reveals fundamental trade‐offs in cell fraction allocation, future investigations incorporating detailed anatomical metrics (e.g., fiber wall thickness, microfibril angle) and direct physiological measurements (e.g., Ks, P50, NSC) are needed to fully elucidate the mechanistic basis of these structural trade‐offs.

### 5.2 Vertical Stratification Induces Shifts in Trade‐Offs Among Xylem Cell Types

The results of the SMA analysis indicate distinct patterns in how the trade‐offs between fiber and vessel fraction and between fiber and parenchyma fraction vary across vertical strata (Figure 4; Table 1; Table 2). For the fiber—vessel trade‐offs, the slopes were consistent across strata, but the intercept of the relationship was significantly higher in the canopy than in the understory (Table 2). In contrast, the fiber—parenchyma trade‐offs showed significantly different slopes across vertical strata (Table 2). This divergence in both intercept and slope strongly suggests a fundamental differentiation in functional strategies between canopy and understory trees, likely driven by the distinct resource demands and ecological constraints inherent to their positions within the vertical forest structure.

Our results show a stronger fiber and vessel fraction trade‐off in canopy trees, characterized by a higher *R* ^2^ with a consistent slope but significantly elevated intercept (Table 1; Table 2). This indicates that for any given fiber fraction, canopy trees consistently allocate a greater proportion to vessel fraction than understory trees (Figure 4; Table 2). This trade‐off pattern in canopy trees is likely functionally associated with demand for hydraulic efficiency (Koch et al. 2004; Mao et al. 2023). Previous research has shown that taller trees tend to develop wider vessels (as also observed in our case, shown in Figure S3) to maintain hydraulic conductivity under the increased gravitational stress and longer hydraulic path lengths (Koch et al. 2004; Stovall et al. 2019; Fajardo et al. 2020; Wu et al. 2025). Concurrently, the elevated intercept further suggests that canopy trees prioritize vessel allocation over a wide range of fiber fractions (Table 2), likely to offset the acute hydraulic constraints imposed by tree height. The stronger trade‐off between fiber and vessel fractions (higher *R* ^2^) observed in canopy trees reflects a tight coupling in the allocation to these two cell types (Table 1). This can be explained by the concurrent need in canopy trees to meet high hydraulic demands while also reinforcing mechanical support for their larger crowns (Pratt and Jacobsen 2017). Consequently, compared to understory trees, canopy trees face a more acute trade‐off between fiber and vessel fractions, reflecting the critical balance they must strike between mechanical support and efficient water transport (Figure 4a).

The fiber‐parenchyma relationship showed a significant difference in slope across vertical strata, indicating a fundamental shift in the nature of this trade‐off (Table 2). The canopy's shallower slope (−0.538) implies that a 1% decrease in fiber fraction corresponds to only an approximately 0.5% increase in parenchyma fraction, whereas the understory's steeper slope (−0.988) indicates a nearly “one‐to‐one” substitution. This steeper slope, coupled with a higher *R* ^2^ value in the understory, suggests that understory trees exhibit a tighter and more constrained trade‐off (Table 1). Unlike canopy trees, which prioritize optimizing fiber‐vessel fractions trade‐off for hydraulic and mechanical demands under ample light resources (Liang 2019), understory trees experience reduced hydraulic constraints due to shorter hydraulic pathways. This shifts the primary selective pressure to the fundamental trade‐off between parenchyma and fiber fractions, leading to two contrasting history strategies of understory trees. Shade‐avoidance trees favor rapid growth by maintaining a lower fiber fraction, which reduces the structural carbon costs of trunk construction and potentially accelerates growth rates (Larjavaara and Muller‐Landau 2010; Poorter et al. 2010; Castorena et al. 2022; González‐Melo 2022; González‐Melo et al. 2025). Furthermore, multiple studies have reported a strong positive correlation between parenchyma fraction and NSC content, indicating that a greater fraction of parenchyma corresponds to a heightened capacity for NSC storage (Plavcová et al. 2016; Kawai et al. 2022; Zhang, Mao, et al. 2022). The increased parenchyma fraction may facilitate NSC storage and translocation, supporting faster growth to acquire more resources in shade‐avoidance trees (Woodcock and Shier 2002; Chen et al. 2017). In contrast, shade‐tolerant trees exhibit slow growth rates, allowing greater allocation of xylem space to fibers, which increases wood density and hardness to better prevent diseases and damage, potentially improving survival rates (Putz et al. 1983; Chen et al. 2017; Oladi et al. 2025). Therefore, understory trees face a stronger trade‐off between growth and defense, which is manifested as the stronger trade‐off between fiber and parenchyma fractions observed in our study (Figure 4b). Nevertheless, the functional linkage to storage and defense, while consistent with anatomical patterns, awaits validation through direct physiological measurements.

Collectively, our findings demonstrate that trade‐off relationships between vessel—fiber and parenchyma—fiber fractions shift across different vertical strata, highlighting how vertical stratification drives divergent spatial allocation patterns among xylem cell types in angiosperm trees. Thus, future investigations of xylem cell‐type trade‐offs must account for these stratum‐specific variations to fully understand tree adaptation strategies in vertically structured forests.

## 6 Conclusion

In this study, we investigated trade‐offs between fiber‐vessel and fiber‐parenchyma in the wood xylem of 39 angiosperm species in eastern China. Our results indicate that canopy trees exhibit a stronger fiber and vessel trade‐off, characterized by a common slope but a significantly higher intercept. In contrast, understory trees demonstrate a stronger fiber‐parenchyma trade‐off and display a steeper slope. Collectively, these findings highlight how vertical stratification drives divergent allocation patterns among xylem cell types, providing new insights into xylem structure of trees across different vertical strata and helping enhance our understanding of their behaviors at different vertical strata. Consequently, future investigations into xylem anatomy need to account for these stratum‐specific variations to fully understand tree adaptation strategies in vertically structured forests.

## Author Contributions

**Qihang Yang:** data curation (equal), investigation (equal), writing – original draft (lead), writing – review and editing (equal). **Yuxin Hong:** data curation (equal), investigation (equal), writing – review and editing (equal). **Hugh Morris:** writing – review and editing (equal). **Faguang Pu:** investigation (equal). **Zuhua Song:** investigation (equal). **Xijin Zhang:** investigation (equal), writing – review and editing (equal). **Kun Song:** funding acquisition (lead), methodology (equal), writing – review and editing (equal).

## Funding

This work was supported by National Natural Science Foundation of China, 31500355.

## Conflicts of Interest

The authors declare no conflicts of interest.

## Supporting information


**Appendix S1:** ece372916‐sup‐0001‐AppendixS1.R.

**Appendix S2:** ece372916‐sup‐0002‐AppendixS2.csv.

**Appendix S3:** ece372916‐sup‐0003‐AppendixS3.docx.

## Data Availability

Data and code used in the analyses are available via Mendeley Data: https://doi.org/10.17632/32v833trwy.3.

## References

[ece372916-bib-0001] Angélico, T. S. , C. R. Marcati , S. Rossi , M. R. da Silva , and J. Sonsin‐Oliveira . 2021. “Soil Effects on Stem Growth and Wood Anatomy of Tamboril Are Mediated by Tree Age.” Forests 12, no. 8: 1058. 10.3390/f12081058.

[ece372916-bib-0002] Araújo, I. , B. S. Marimon , B. H. M. Junior , et al. 2024. “Taller Trees Exhibit Greater Hydraulic Vulnerability in Southern Amazonian Forests.” Environmental and Experimental Botany 226: 105905. 10.1016/j.envexpbot.2024.105905.

[ece372916-bib-0003] Aritsara, A. N. A. , V. M. Razakandraibe , T. Ramananantoandro , S. M. Gleason , and K. F. Cao . 2021. “Increasing Axial Parenchyma Fraction in the Malagasy Magnoliids Facilitated the Co‐Optimisation of Hydraulic Efficiency and Safety.” New Phytologist 229, no. 3: 1467–1480. 10.1111/nph.16969. 32981106

[ece372916-bib-0004] Brodersen, C. R. , A. J. McElrone , B. Choat , M. A. Matthews , and K. A. Shackel . 2010. “The Dynamics of Embolism Repair in Xylem: In Vivo Visualizations Using High‐Resolution Computed Tomography.” Plant Physiology 154, no. 3: 1088–1095. 10.1104/pp.110.162396. 20841451 PMC2971590

[ece372916-bib-0005] Castorena, M. , M. E. Olson , B. J. Enquist , and A. Fajardo . 2022. “Toward a General Theory of Plant Carbon Economics.” Trends in Ecology & Evolution 37, no. 10: 829–837. 10.1016/j.tree.2022.05.007. 35717415

[ece372916-bib-0006] Chave, J. , D. Coomes , S. Jansen , S. L. Lewis , N. G. Swenson , and A. E. Zanne . 2009. “Towards a Worldwide Wood Economics Spectrum.” Ecology Letters 12, no. 4: 351–366. 10.1111/j.1461-0248.2009.01285.x. 19243406

[ece372916-bib-0007] Chen, L. , W. Xiang , H. Wu , et al. 2017. “Tree Growth Traits and Social Status Affect the Wood Density of Pioneer Species in Secondary Subtropical Forest.” Ecology and Evolution 7, no. 14: 5366–5377. 10.1002/ece3.3110. 28770074 PMC5528239

[ece372916-bib-0008] Choat, B. , A. R. Cobb , and S. Jansen . 2008. “Structure and Function of Bordered Pits: New Discoveries and Impacts on Whole‐Plant Hydraulic Function.” New Phytologist 177, no. 3: 608–626. 10.1111/j.1469-8137.2007.02317.x. 18086228

[ece372916-bib-0009] Deflorio, G. , C. Johnson , S. Fink , and F. W. M. R. Schwarze . 2008. “Decay Development in Living Sapwood of Coniferous and Deciduous Trees Inoculated With Six Wood Decay Fungi.” Forest Ecology and Management 255, no. 7: 2373–2383. 10.1016/j.foreco.2007.12.040.

[ece372916-bib-0010] Dória, L. C. , J. Sonsin‐Oliveira , S. Rossi , and C. R. Marcati . 2022. “Functional Trade‐Offs in Volume Allocation to Xylem Cell Types in 75 Species From the Brazilian Savanna Cerrado.” Annals of Botany 130, no. 3: 445–456. 10.1093/aob/mcac095. 35863898 PMC9486921

[ece372916-bib-0011] Fajardo, A. , C. Martínez‐Pérez , M. A. Cervantes‐Alcayde , and M. E. Olson . 2020. “Stem Length, Not Climate, Controls Vessel Diameter in Two Trees Species Across a Sharp Precipitation Gradient.” New Phytologist 225, no. 6: 2347–2355. 10.1111/nph.16287. 31657018

[ece372916-bib-0012] Flor, L. , G. Toro , M. Carriquí , et al. 2025. “Impact of Severe Water Stress on Drought Resistance Mechanisms and Hydraulic Vulnerability Segmentation in Grapevine: The Role of Rootstock.” Journal of Experimental Botany 76: 3141–3157. 10.1093/jxb/eraf044. 39902836

[ece372916-bib-0013] González‐Melo, A. 2022. “Wood Anatomical Traits Mediate Life‐History Variations at the Sapling, but Not at the Adult Stage.” Trees 36, no. 4: 1337–1347. 10.1007/s00468-022-02293-1.

[ece372916-bib-0014] González‐Melo, A. , B. Salgado‐Negret , N. Norden , et al. 2025. “Linking Seedling Wood Anatomical Trade‐Offs With Drought and Seedling Growth and Survival in Tropical Dry Forests.” New Phytologist 245, no. 1: 117–129. 10.1111/nph.20222. 39473120 PMC11617663

[ece372916-bib-0015] Hacke, U. G. , J. S. Sperry , W. T. Pockman , S. D. Davis , and K. A. McCulloh . 2001. “Trends in Wood Density and Structure Are Linked to Prevention of Xylem Implosion by Negative Pressure.” Oecologia 126, no. 4: 457–461. 10.1007/s004420100628. 28547229

[ece372916-bib-0016] Hellmann, E. , D. Ko , R. Ruonala , and Y. Helariutta . 2018. “Plant Vascular Tissues—Connecting Tissue Comes in All Shapes.” Plants 7, no. 4: 109. 10.3390/plants7040109. 30551673 PMC6313914

[ece372916-bib-0017] Hoch, G. , A. Richter , and C. Körner . 2003. “Non‐Structural Carbon Compounds in Temperate Forest Trees.” Plant, Cell and Environment 26, no. 7: 1067–1081. 10.1046/j.0016-8025.2003.01032.x.

[ece372916-bib-0018] Janssen, T. A. , T. Hölttä , K. Fleischer , K. Naudts , and H. Dolman . 2020. “Wood Allocation Trade‐Offs Between Fiber Wall, Fiber Lumen, and Axial Parenchyma Drive Drought Resistance in Neotropical Trees.” Plant, Cell and Environment 43, no. 4: 965–980. 10.1111/pce.13687. PMC7155043 31760666

[ece372916-bib-0019] Kawai, K. , K. Minagi , T. Nakamura , S.‐T. Saiki , K. Yazaki , and A. Ishida . 2022. “Parenchyma Underlies the Interspecific Variation of Xylem Hydraulics and Carbon Storage Across 15 Woody Species on a Subtropical Island in Japan.” Tree Physiology 42, no. 2: 337–350. 10.1093/treephys/tpab100. 34328187

[ece372916-bib-0020] Kiorapostolou, N. , L. Da Sois , F. Petruzzellis , et al. 2019. “Vulnerability to Xylem Embolism Correlates to Wood Parenchyma Fraction in Angiosperms but Not in Gymnosperms.” Tree Physiology 39, no. 10: 1675–1684. 10.1093/treephys/tpz068. 31211372

[ece372916-bib-0021] Koch, G. W. , S. C. Sillett , G. M. Jennings , and S. D. Davis . 2004. “The Limits to Tree Height.” Nature 428, no. 6985: 851–854. 10.1038/nature02417. 15103376

[ece372916-bib-0022] Larjavaara, M. , and H. C. Muller‐Landau . 2010. “Rethinking the Value of High Wood Density.” Functional Ecology 24, no. 4: 701–705. 10.1111/j.1365-2435.2010.01698.x.

[ece372916-bib-0023] Lechthaler, S. , N. Kiorapostolou , A. Pitacco , T. Anfodillo , and G. Petit . 2020. “The Total Path Length Hydraulic Resistance According to Known Anatomical Patterns: What Is the Shape of the Root‐To‐Leaf Tension Gradient Along the Plant Longitudinal Axis?” Journal of Theoretical Biology 502: 110369. 10.1016/j.jtbi.2020.110369. 32526220

[ece372916-bib-0024] Legendre, P. , and M. J. Oksanen . 2018. “Lmodel2: Model ii Regression. R Package Version 1.7–3.”

[ece372916-bib-0025] Lens, F. , S. M. Gleason , G. Bortolami , C. Brodersen , S. Delzon , and S. Jansen . 2022. “Functional Xylem Characteristics Associated With Drought‐Induced Embolism in Angiosperms.” New Phytologist 236, no. 6: 2019–2036. 10.1111/nph.18447. 36039697

[ece372916-bib-0026] Lens, F. , J. S. Sperry , M. A. Christman , B. Choat , D. Rabaey , and S. Jansen . 2011. “Testing Hypotheses That Link Wood Anatomy to Cavitation Resistance and Hydraulic Conductivity in the Genus Acer.” New Phytologist 190, no. 3: 709–723. 10.1111/j.1469-8137.2010.03518.x. 21054413

[ece372916-bib-0027] Liang, S. 2019. “Vegetation Height and Vertical Structure.” In Advanced Remote Sensing, edited by S. Liang and J. Wang , 511–542. Elsevier Science and Technology. 10.1016/B978-0-12-815826-5.00013-1.

[ece372916-bib-0028] Liu, H. , S. Gleason , G. Hao , et al. 2019. “Hydraulic Traits Are Coordinated With Maximum Plant Height at the Global Scale.” Science Advances 5, no. 2: eaav1332. 10.1126/sciadv.aav1332. 30788435 PMC6374111

[ece372916-bib-0029] Liu, K. , J. Cao , S. Zhou , and F. Pu . 2016. “The Floristic Analysis of Seed Plants in An Hui Tian Ma National Nature Reserve in Jin Zhai.” Journal of West Anhui University 2: 12–15.

[ece372916-bib-0030] Mao, J. , Y. Luo , C. Jin , M. Xu , X. Li , and Y. Tian . 2023. “Response of Leaf Photosynthesis–Transpiration Coupling to Biotic and Abiotic Factors in the Typical Desert Shrub *Artemisia ordosica* .” Sustainability 15, no. 13: 10216. 10.3390/su151310216.

[ece372916-bib-0031] Martínez‐Cabrera, H. I. , C. S. Jones , S. Espino , and H. J. Schenk . 2009. “Wood Anatomy and Wood Density in Shrubs: Responses to Varying Aridity Along Transcontinental Transects.” American Journal of Botany 96, no. 8: 1388–1398. 10.3732/ajb.0800237. 21628286

[ece372916-bib-0032] Morris, H. , M. A. Gillingham , L. Plavcova , et al. 2018. “Vessel Diameter Is Related to Amount and Spatial Arrangement of Axial Parenchyma in Woody Angiosperms.” Plant, Cell & Environment 41, no. 1: 245–260. 10.1111/pce.13091. 29047119

[ece372916-bib-0033] Morris, H. , L. Plavcová , P. Cvecko , et al. 2016. “A Global Analysis of Parenchyma Tissue Fractions in Secondary Xylem of Seed Plants.” New Phytologist 209, no. 4: 1553–1565. 10.1111/nph.13737. 26551018 PMC5063116

[ece372916-bib-0034] Oladi, R. , R. Aliverdikhani , and E. Abdi . 2025. “Linking Root Xylem Anatomy to Tensile Strength: Insights From Four Broadleaved Tree Species in the Hyrcanian Forests.” Plant and Soil 512, no. 1: 1311–1326. 10.1007/s11104-024-07148-x.

[ece372916-bib-0035] Olson, M. E. , D. Soriano , J. A. Rosell , et al. 2018. “Plant Height and Hydraulic Vulnerability to Drought and Cold.” Proceedings of the National Academy of Sciences of the United States of America 115, no. 29: 7551–7556. 10.1073/pnas.1721728115. 29967148 PMC6055177

[ece372916-bib-0036] Onoda, Y. , A. E. Richards , and M. Westoby . 2010. “The Relationship Between Stem Biomechanics and Wood Density Is Modified by Rainfall in 32 Australian Woody Plant Species.” New Phytologist 185, no. 2: 493–501. 10.1111/j.1469-8137.2009.03088.x. 19925557

[ece372916-bib-0037] Petit, G. , M. Mencuccini , M. Carrer , A. L. Prendin , and T. Hölttä . 2023. “Axial Conduit Widening, Tree Height, and Height Growth Rate Set the Hydraulic Transition of Sapwood Into Heartwood.” Journal of Experimental Botany 74, no. 17: 5072–5087. 10.1093/jxb/erad227. 37352139

[ece372916-bib-0038] Phillips, N. , M. Ryan , B. Bond , N. McDowell , T. Hinckley , and J. Čermák . 2003. “Reliance on Stored Water Increases With Tree Size in Three Species in the Pacific Northwest.” Tree Physiology 23, no. 4: 237–245. 10.1093/treephys/23.4.237. 12566259

[ece372916-bib-0039] Plavcová, L. , F. Gallenmüller , H. Morris , M. Khatamirad , S. Jansen , and T. Speck . 2019. “Mechanical Properties and Structure–Function Trade‐Offs in Secondary Xylem of Young Roots and Stems.” Journal of Experimental Botany 70, no. 14: 3679–3691. 10.1093/jxb/erz286. 31301134

[ece372916-bib-0040] Plavcová, L. , G. Hoch , H. Morris , S. Ghiasi , and S. Jansen . 2016. “The Amount of Parenchyma and Living Fibers Affects Storage of Nonstructural Carbohydrates in Young Stems and Roots of Temperate Trees.” American Journal of Botany 103, no. 4: 603–612. 10.3732/ajb.1500489. 26993972

[ece372916-bib-0041] Plavcová, L. , V. Jandová , J. Altman , P. Liancourt , K. Korznikov , and J. Doležal . 2024. “Variations in Wood Anatomy in Afrotropical Trees With a Particular Emphasis on Radial and Axial Parenchyma.” Annals of Botany 134, no. 1: 151–162. 10.1093/aob/mcae049. 38525918 PMC11161563

[ece372916-bib-0042] Poorter, L. , I. McDonald , A. Alarcón , et al. 2010. “The Importance of Wood Traits and Hydraulic Conductance for the Performance and Life History Strategies of 42 Rainforest Tree Species.” New Phytologist 185, no. 2: 481–492. 10.1111/j.1469-8137.2009.03092.x. 19925555

[ece372916-bib-0043] Pratt, R. , A. Jacobsen , F. Ewers , and S. Davis . 2007. “Relationships Among Xylem Transport, Biomechanics and Storage in Stems and Roots of Nine Rhamnaceae Species of the California Chaparral.” New Phytologist 174, no. 4: 787–798. 10.1111/j.1469-8137.2007.02061.x. 17504462

[ece372916-bib-0044] Pratt, R. , A. Jacobsen , M. Percolla , M. De Guzman , C. Traugh , and M. Tobin . 2021. “Trade‐Offs Among Transport, Support, and Storage in Xylem From Shrubs in a Semiarid Chaparral Environment Tested With Structural Equation Modeling.” Proceedings of the National Academy of Sciences of the United States of America 118, no. 33: e2104336118. 10.1073/pnas.2104336118. 34389676 PMC8379947

[ece372916-bib-0045] Pratt, R. B. , and A. L. Jacobsen . 2017. “Conflicting Demands on Angiosperm Xylem: Tradeoffs Among Storage, Transport and Biomechanics.” Plant, Cell & Environment 40, no. 6: 897–913. 10.1111/pce.12862. 27861981

[ece372916-bib-0046] Pratt, R. B. , M. F. Tobin , A. L. Jacobsen , et al. 2021. “Starch Storage Capacity of Sapwood Is Related to Dehydration Avoidance During Drought.” American Journal of Botany 108, no. 1: 91–101. 10.1002/ajb2.1586. 33349932

[ece372916-bib-0047] Preston, K. A. , W. K. Cornwell , and J. L. DeNoyer . 2006. “Wood Density and Vessel Traits as Distinct Correlates of Ecological Strategy in 51 California Coast Range Angiosperms.” New Phytologist 170, no. 4: 807–818. 10.1111/j.1469-8137.2006.01712.x. 16684240

[ece372916-bib-0048] Putz, F. E. , P. D. Coley , K. Lu , A. Montalvo , and A. Aiello . 1983. “Uprooting and Snapping of Trees: Structural Determinants and Ecological Consequences.” Canadian Journal of Forest Research 13, no. 5: 1011–1020. 10.1139/x83-133.

[ece372916-bib-0049] Rodriguez‐Zaccaro, F. D. , J. Valdovinos‐Ayala , M. I. Percolla , M. D. Venturas , R. B. Pratt , and A. L. Jacobsen . 2019. “Wood Structure and Function Change With Maturity: Age of the Vascular Cambium Is Associated With Xylem Changes in Current‐Year Growth.” Plant, Cell & Environment 42, no. 6: 1816–1831. 10.1111/pce.13528. 30707440

[ece372916-bib-0050] Ryan, M. G. , N. Phillips , and B. J. Bond . 2006. “The Hydraulic Limitation Hypothesis Revisited.” Plant, Cell and Environment 29, no. 3: 367–381. 10.1111/j.1365-3040.2005.01478.x. 17080592

[ece372916-bib-0051] Ryan, M. G. , and B. J. Yoder . 1997. “Hydraulic Limits to Tree Height and Tree Growth.” Bioscience 47, no. 4: 235–242. 10.2307/1313077.

[ece372916-bib-0052] Schneider, L. , and H. Gärtner . 2013. “The Advantage of Using a Starch Based Non‐Newtonian Fluid to Prepare Micro Sections.” Dendrochronologia 31, no. 3: 175–178. 10.1016/j.dendro.2013.04.002.

[ece372916-bib-0053] Słupianek, A. , A. Dolzblasz , and K. Sokołowska . 2021. “Xylem Parenchyma—Role and Relevance in Wood Functioning in Trees.” Plants 10, no. 6: 1247. 10.3390/plants10061247. 34205276 PMC8235782

[ece372916-bib-0054] Sperry, J. S. , and U. G. Hacke . 2004. “Analysis of Circular Bordered Pit Function i. Angiosperm Vessels With Homogenous Pit Membranes.” American Journal of Botany 91, no. 3: 369–385. 10.3732/ajb.91.3.369. 21653393

[ece372916-bib-0055] Sperry, J. S. , U. G. Hacke , and J. Pittermann . 2006. “Size and Function in Conifer Tracheids and Angiosperm Vessels.” American Journal of Botany 93, no. 10: 1490–1500. 10.3732/ajb.93.10.1490. 21642096

[ece372916-bib-0056] Sperry, J. S. , F. C. Meinzer , and K. A. McCULLOH . 2008. “Safety and Efficiency Conflicts in Hydraulic Architecture: Scaling From Tissues to Trees.” Plant, Cell & Environment 31, no. 5: 632–645. 10.1111/j.1365-3040.2007.01765.x. 18088335

[ece372916-bib-0057] Spicer, R. 2014. “Symplasmic Networks in Secondary Vascular Tissues: Parenchyma Distribution and Activity Supporting Long‐Distance Transport.” Journal of Experimental Botany 65, no. 7: 1829–1848. 10.1093/jxb/ert459. 24453225

[ece372916-bib-0058] Stovall, A. E. , H. Shugart , and X. Yang . 2019. “Tree Height Explains Mortality Risk During an Intense Drought.” Nature Communications 10, no. 1: 4385. 10.1038/s41467-019-12380-6. PMC6763443 31558795

[ece372916-bib-0059] Tricerri, N. , M. Tomasella , S. Cavalletto , et al. 2025. “Fibers Beyond Structure: Do They Contribute to Embolism Reversal After Drought Relief in Poplar?” New Phytologist 247, no. 2: 612–624. 10.1111/nph.70179. 40313028 PMC12177288

[ece372916-bib-0060] Tyree, M. T. , and F. W. Ewers . 1991. “The Hydraulic Architecture of Trees and Other Woody Plants.” New Phytologist 119, no. 3: 345–360. 10.1111/j.1469-8137.1991.tb00035.x.

[ece372916-bib-0061] Tyree, M. T. , and S. Yang . 1990. “Water‐Storage Capacity of Thuja, Tsuga and Acer Stems Measured by Dehydration Isotherms: The Contribution of Capillary Water and Cavitation.” Planta 182, no. 3: 420–426. 10.1007/BF02411394. 24197194

[ece372916-bib-0062] Warton, D. I. , R. A. Duursma , D. S. Falster , and S. Taskinen . 2012. “Smatr 3‐An r Package for Estimation and Inference About Allometric Lines.” Methods in Ecology and Evolution 3, no. 2: 257–259. 10.1111/j.2041-210X.2011.00153.x.

[ece372916-bib-0063] Wheeler, E. A. 2011. “Inside Wood–a Web Resource for Hardwood Anatomy.” IAWA Journal 32, no. 2: 199–211. 10.1163/22941932-90000051.

[ece372916-bib-0064] Woodcock, D. , and A. Shier . 2002. “Wood Specific Gravity and Its Radial Variations: The Many Ways to Make a Tree.” Trees 16, no. 6: 437–443. 10.1007/s00468-002-0173-7.

[ece372916-bib-0065] Wu, D. , X. Zhou , J. Wang , H. Morris , X. Zhang , and K. Song . 2025. “Tree Height and Not Climate Influences Intraspecific Variations in Wood Parenchyma Fractions of Angiosperm Species in a Mountain Forest of Eastern China.” American Journal of Botany 112, no. 5: e70035. 10.1002/ajb2.70035. 40292570

[ece372916-bib-0066] Xing, S. , L. Leahy , L. A. Ashton , R. L. Kitching , T. C. Bonebrake , and B. R. Scheffers . 2023. “Ecological Patterns and Processes in the Vertical Dimension of Terrestrial Ecosystems.” Journal of Animal Ecology 92, no. 3: 538–551. 10.1111/1365-2656.13881. 36622247

[ece372916-bib-0067] Zhang, G. , Z. Mao , C. Fortunel , et al. 2022. “Parenchyma Fractions Drive the Storage Capacity of Nonstructural Carbohydrates Across a Broad Range of Tree Species.” American Journal of Botany 109, no. 4: 535–549. 10.1002/ajb2.1838. 35266560

[ece372916-bib-0068] Zhang, G. , Z. Mao , P. Maillard , et al. 2023. “Functional Trade‐Offs Are Driven by Coordinated Changes Among Cell Types in the Wood of Angiosperm Trees From Different Climates.” New Phytologist 240, no. 3: 1162–1176. 10.1111/nph.19132. 37485789

[ece372916-bib-0069] Zhang, K. Y. , D. Yang , Y. B. Zhang , et al. 2024. “Linkages Among Stem Xylem Transport, Biomechanics, and Storage in Lianas and Trees Across Three Contrasting Environments.” American Journal of Botany 111, no. 3: e16290. 10.1002/ajb2.16290. 38380953

[ece372916-bib-0070] Zhang, X. , Q. Li , Y. Yang , et al. 2024. “A Higher Tissue Fraction of Parenchyma in Secondary Xylem Supports Growth Recovery of Angiosperm Trees After Drought.” Functional Ecology 38, no. 12: 2709–2719. 10.1111/1365-2435.14680.

[ece372916-bib-0071] Zhang, X. , D. Wu , Q. Li , et al. 2022. “Influence of Colder Temperature on the Axial and Radial Parenchyma Fraction of *Quercus ciliaris* CC Huang & YT Chang Wood and Its Relationship With Carbohydrate Reserve (NSC).” Forests 13, no. 2: 169. 10.3390/f13020169.

[ece372916-bib-0072] Zheng, J. , and H. I. Martínez‐Cabrera . 2013. “Wood Anatomical Correlates With Theoretical Conductivity and Wood Density Across China: Evolutionary Evidence of the Functional Differentiation of Axial and Radial Parenchyma.” Annals of Botany 112, no. 5: 927–935. 10.1093/aob/mct153. 23904446 PMC3747806

[ece372916-bib-0073] Zheng, J. , X. Zhao , H. Morris , and S. Jansen . 2019. “Phylogeny Best Explains Latitudinal Patterns of Xylem Tissue Fractions for Woody Angiosperm Species Across China.” Frontiers in Plant Science 10: 556. 10.3389/fpls.2019.00556. 31130973 PMC6509232

[ece372916-bib-0074] Ziemińska, K. , D. W. Butler , S. M. Gleason , I. J. Wright , and M. Westoby . 2013. “Fibre Wall and Lumen Fractions Drive Wood Density Variation Across 24 Australian Angiosperms.” AoB Plants 5: plt046. 10.1093/aobpla/plt046.

[ece372916-bib-0075] Ziemińska, K. , E. Rosa , S. M. Gleason , and N. M. Holbrook . 2020. “Wood Day Capacitance Is Related to Water Content, Wood Density, and Anatomy Across 30 Temperate Tree Species.” Plant, Cell and Environment 43, no. 12: 3048–3067. 10.1111/pce.13891. 32935340

