# Supplementary material for: Vertical Stratification Drives Divergent Spatial Trade‐Offs Among Xylem Cell Types in Angiosperm Trees of a Mountain Forest in Eastern China
Source: Ecol Evol. 2026 Jan 7;16(1):e72916. doi: 10.1002/ece3.72916 (PMC12779537; doi:10.1002/ece3.72916)
Supplement: Supplementary file 3 — Appendix S3: ece372916‐sup‐0003‐AppendixS3.docx. [file ECE3-16-e72916-s001.docx]

**Supporting Materials and Methods**

Vessel density was calculated as the ratio of the number of vessel individuals within the annotated region to the area of the annotated region. Assuming circular vessel shapes, the average vessel diameter (*V_d_*) was obtained from the vessel area, calculated using the following formula,

$$V_{d}= \frac{\sum\sqrt{\frac{4S}{\pi}}}{n}$$

where *n* is the number of vessels and *S* is the area of individual vessels.


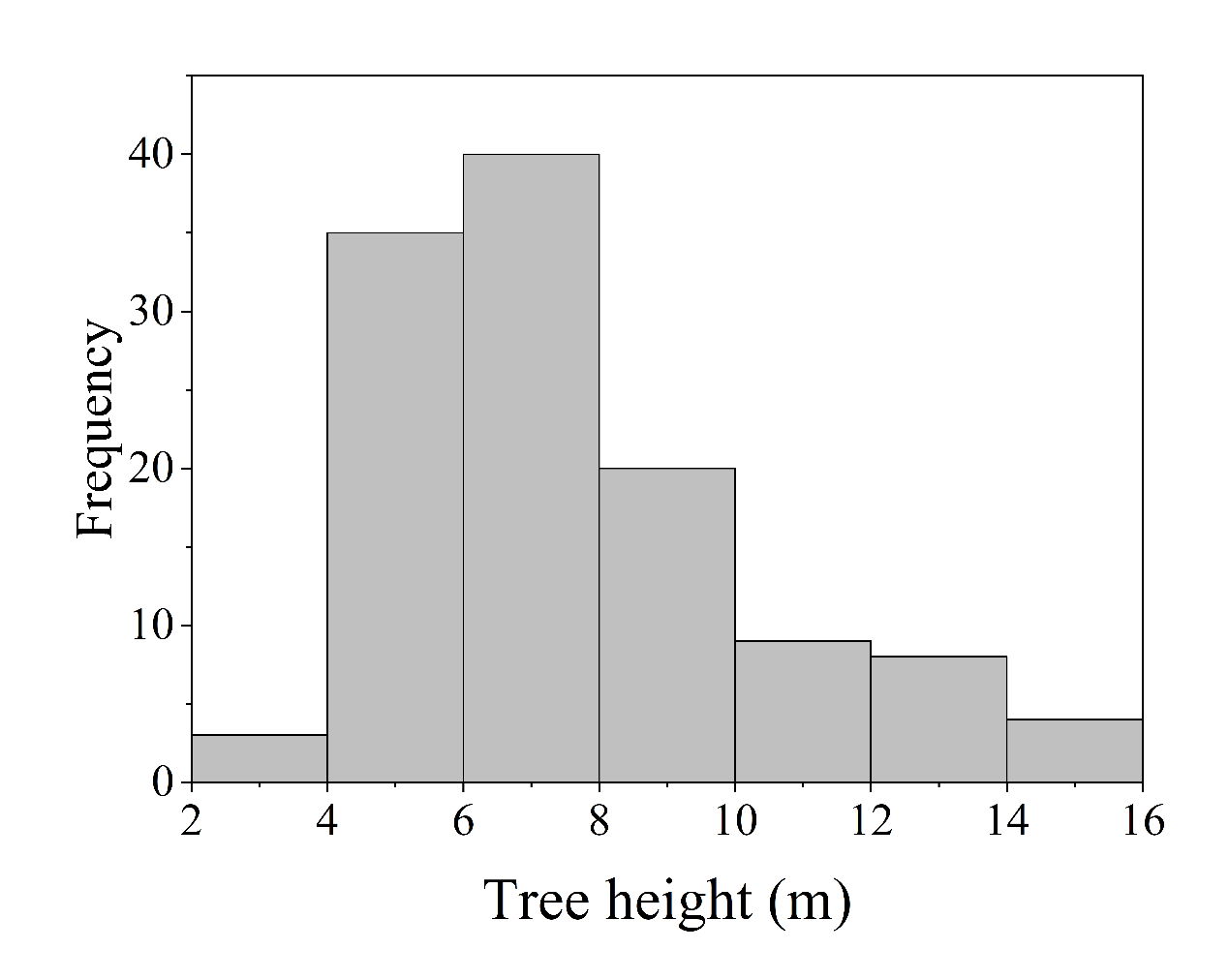


**Figure S1** Frequency distribution histogram of tree heights for 119 individual trees.


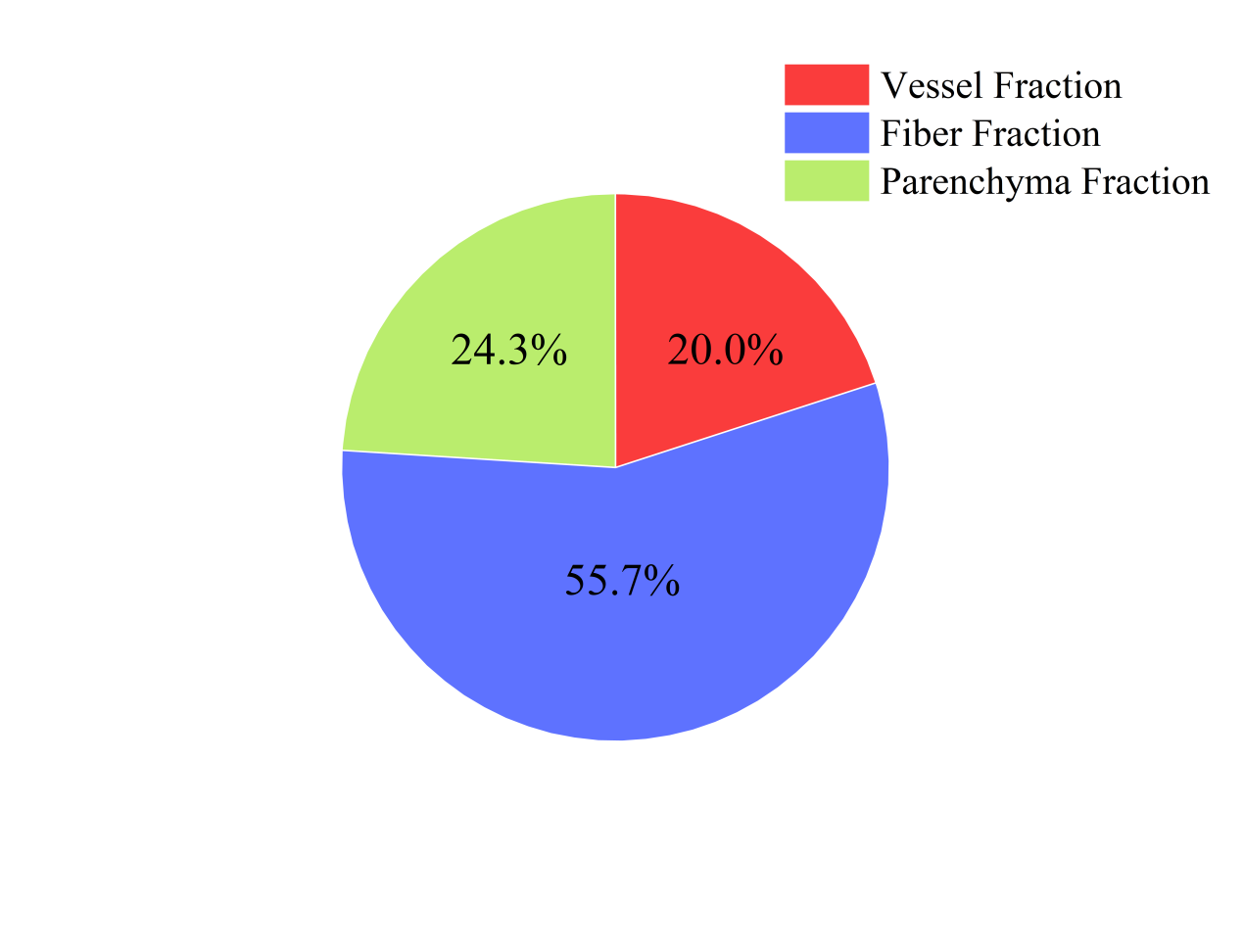


**Figure S2** Mean volumetric fractions of different cell types in wood across 119 individuals.

**
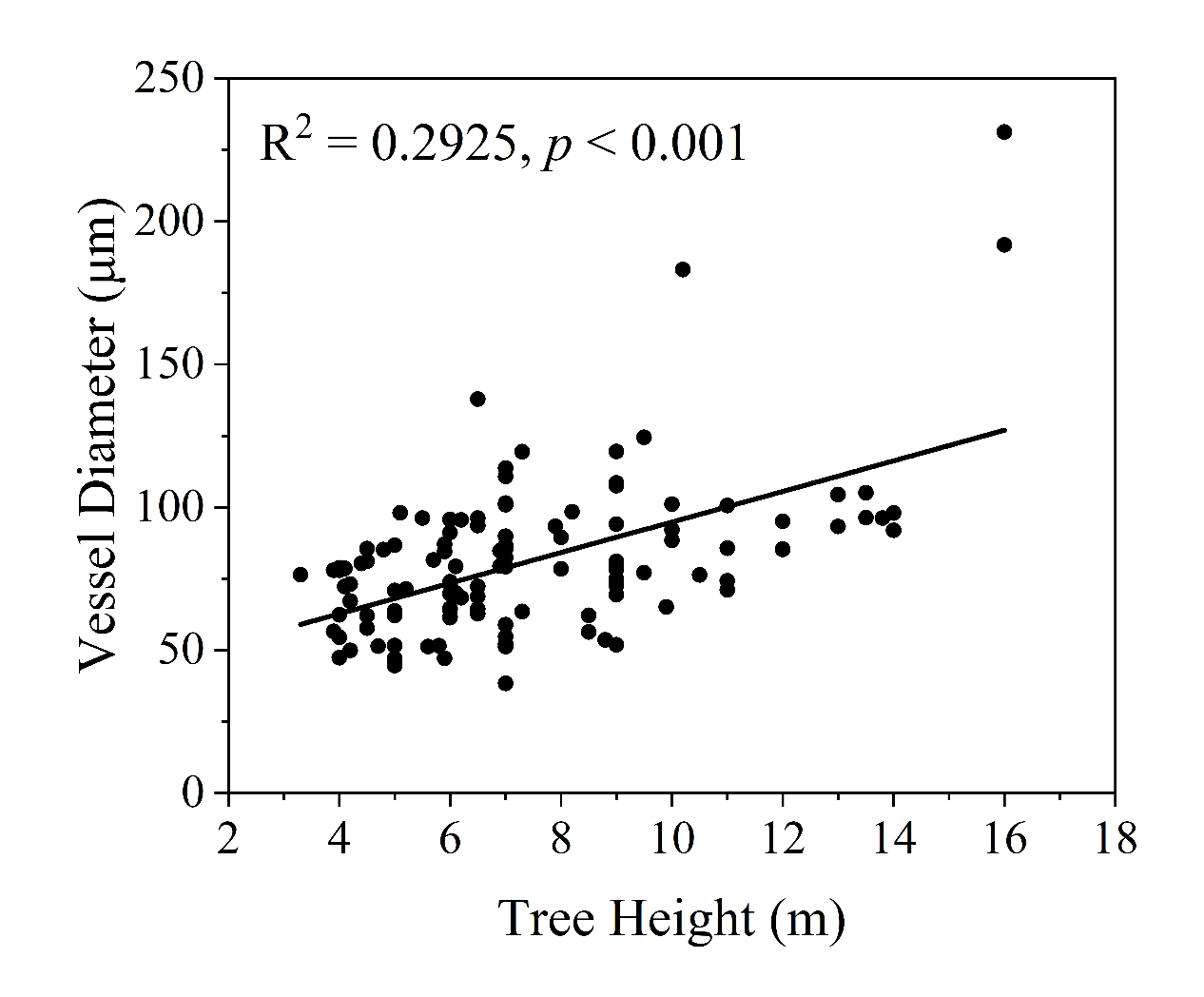
**

**Figure S3** Simple linear regression analysis of vessel diameter and tree height. Significant regression line, R^2^, and *p*-value are shown.

**
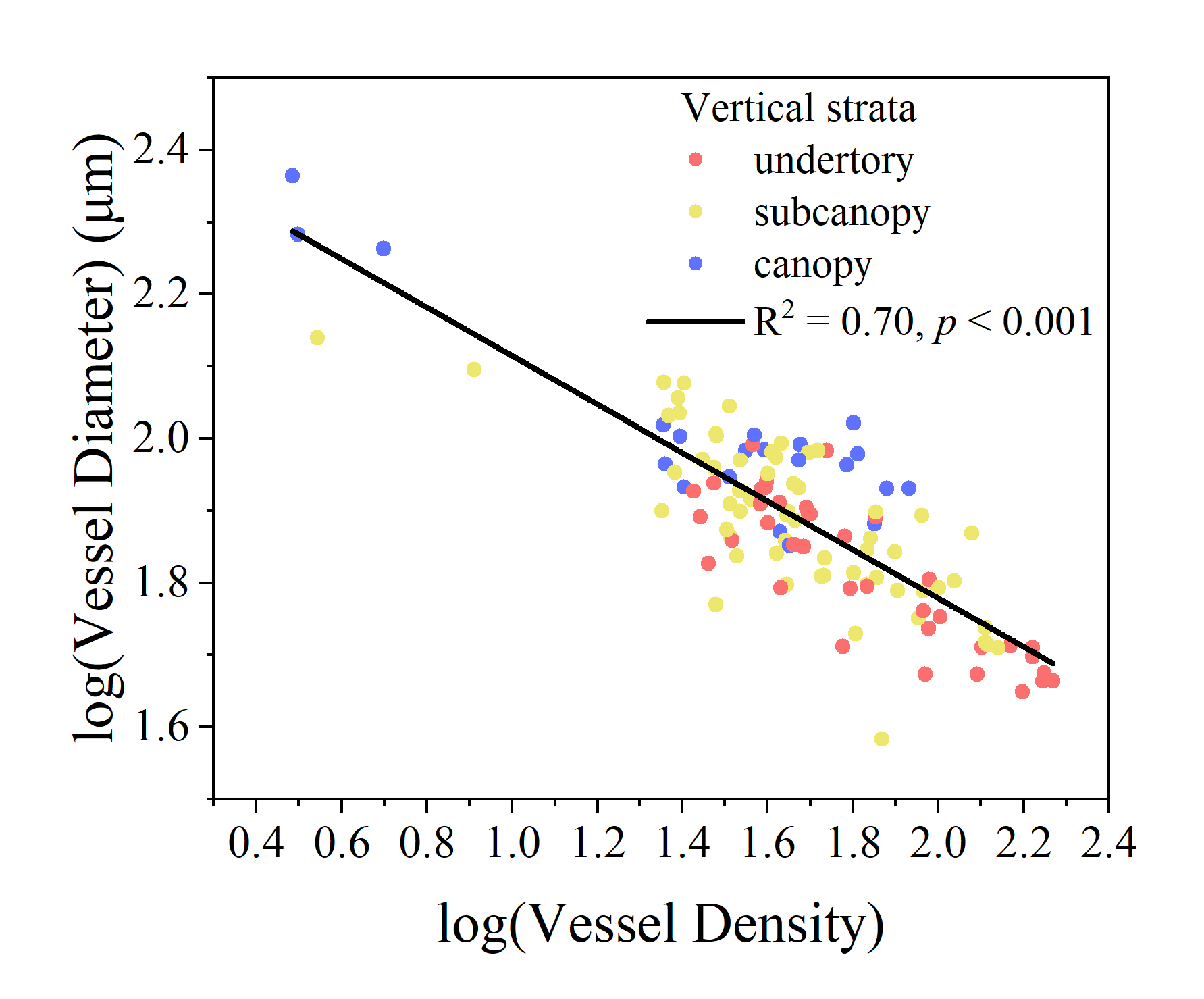
**

**Figure S4** Simple linear regression analysis of log-transformed vessel diameter and log-transformed vessel density. Significant regression line, R^2^, and *p*-value are shown. Different colors represent individuals from different vertical strata.

**Table S1** Summary of sampled species for each height class.

| Vertical strata | species | individuals |
| --- | --- | --- |
| understory | *Symplocos tanakana* | 6 |
|  | *Photinia megaphylla* | 1 |
|  | *Cornus controversa* | 1 |
|  | *Carpinus turczaninowii* | 3 |
|  | *Viburnum sympodiale* | 1 |
|  | *Daphniphyllum macropodum* | 1 |
|  | *Hamamelis mollis* | 2 |
|  | *Corylopsis sinensis* | 3 |
|  | *Meliosma veitchiorum* | 1 |
|  | *Carpinus cordata* | 2 |
|  | *Acer davidii* | 1 |
|  | *Lindera glauca* | 2 |
|  | *Sorbus alnifolia* | 3 |
|  | *Cornus kousa* subsp. *chinensis* | 5 |
|  | *Prunus dielsiana* | 1 |
|  | *Photinia parvifolia* | 1 |
|  | *Styrax japonicus* | 1 |
|  | *Prunus pseudocerasus* | 1 |
|  | *Camellia oleifera* | 2 |
| subcanopy | *Symplocos tanakana* | 6 |
|  | *Ilex macropoda* | 5 |
|  | *Lindera praecox* | 1 |
|  | *Cornus controversa* | 4 |
|  | *Ilex chinensis* | 1 |
|  | *Carpinus turczaninowii* | 2 |
|  | *Acer davidii* subsp. *grosseri* | 1 |
|  | *Photinia hirsuta* | 1 |
|  | *Malus hupehensis* | 1 |
|  | *Tilia japonica* | 1 |
|  | *Acer palmatum* | 1 |
|  | *Corylopsis sinensis* | 3 |
|  | *Carpinus viminea* | 1 |
|  | *Tetradium glabrifolium* | 1 |
|  | *Maackia hupehensis* | 1 |
|  | *Acer davidii* | 3 |
|  | *Sorbus folgneri* | 1 |
|  | *Sorbus alnifolia* | 2 |
|  | *Cornus kousa* subsp. *chinensis* | 2 |
|  | *Prunus dielsiana* | 2 |
|  | *Pterostyrax corymbosus* | 3 |
|  | *Prunus pseudocerasus* | 8 |
|  | *Styrax obassia* | 1 |
|  | *Photinia beauverdiana* | 2 |
|  | *Stewartia sinensis* | 6 |
| canopy | *Symplocos tanakana* | 1 |
|  | *Ilex macropoda* | 1 |
|  | *Cornus controversa* | 1 |
|  | *Carpinus turczaninowii* | 1 |
|  | *Liquidambar formosana* | 6 |
|  | *Pterocarya stenoptera* | 2 |
|  | *Diospyros lotus* | 1 |
|  | *Carpinus viminea* | 1 |
|  | *Meliosma veitchiorum* | 2 |
|  | *Prunus obtusata* | 1 |
|  | *Pterostyrax corymbosus* | 2 |
|  | *Prunus pseudocerasus* | 1 |
|  | *Stewartia sinensis* | 1 |

**Table S2** Average, minimum, and maximum volumetric fractions of different cell types in wood across 119 individuals.

| Cell Type | Average | Minimum | Maximum |
| --- | --- | --- | --- |
| Fiber Fraction | 0.557 | 0.369 | 0.809 |
| Parenchyma Fraction | 0.243 | 0.044 | 0.401 |
| Vessel Fraction | 0.200 | 0.040 | 0.423 |

**Table S3** Comparisons of linear and quadratic models for whole-dataset and stratified analyses.

| Trade-offs | strata | model types | AIC | *p* value |
| --- | --- | --- | --- | --- |
| Fiber Fraction - Vessel Fraction | understory | linear | -89.36 | 0.896 |
|  |  | quadratic | -87.38 |  |
|  | subcanopy | linear | -127.61 | 0.599 |
|  |  | quadratic | -125.90 |  |
|  | canopy | linear | -51.71 | 0.534 |
|  |  | quadratic | -50.17 |  |
|  | all strata | linear | -269.51 | 0.670 |
|  |  | quadratic | -267.70 |  |
| Fiber Fraction - Total Parenchyma Fraction | understory | linear | -114.76 | 0.013 |
|  |  | quadratic | -119.51 |  |
|  | subcanopy | linear | -159.91 | 0.173 |
|  |  | quadratic | -159.88 |  |
|  | canopy | linear | -29.28 | 0.573 |
|  |  | quadratic | -27.66 |  |
|  | all strata | linear | -294.55 | 0.013 |
|  |  | quadratic | -298.89 |  |

**Table S4** Results of a linear mixed model of the relationship between Vessel - Fiber Fraction and Total Parenchyma -Fiber Fraction for different individuals

| Trade-offs | Fixed effect | Coefficients | Standard error | *p* value | Marginal R^2^/  Conditional R^2^ |
| --- | --- | --- | --- | --- | --- |
| Fiber Fraction - Vessel Fraction | Vessel Fraction | -0.669 | 0.093 | ＜0.001 | 0.257/0.776 |
| Fiber Fraction - Total RAP Fraction | Total RAP Fraction | -0.714 | 0.083 | ＜0.001 | 0.384/0.772 |
